# Trends in Trauma Admissions During the COVID-19 Pandemic in Los Angeles County, California

**DOI:** 10.1001/jamanetworkopen.2021.1320

**Published:** 2021-02-22

**Authors:** Cameron Ghafil, Kazuhide Matsushima, Li Ding, Reynold Henry, Kenji Inaba

**Affiliations:** 1 Division of Acute Care Surgery, University of Southern California, Los Angeles; 2 Department of Preventive Medicine, University of Southern California, Los Angeles

## Abstract

**Importance:**

Describing the changes in trauma volume and injury patterns during the course of the coronavirus disease 2019 (COVID-19) pandemic could help to inform policy development and hospital resource planning.

**Objective:**

To examine trends in trauma admissions throughout Los Angeles County (LAC) during the pandemic.

**Design, Setting, and Participants:**

In this cohort study, all trauma admissions to the 15 verified level 1 and level 2 trauma centers in LAC from January 1 to June 7, 2020 were reviewed. All trauma admissions from the same period in 2019 were used as historical control. For overall admissions, the study period was divided into 3 intervals based on daily admission trend analysis (January 1 through February 28, March 1 through April 9, April 10 through June 7). For the blunt trauma subgroup analysis, the study period was divided into 3 similar intervals (January 1 through February 27, February 28 through April 5, April 6 through June 7).

**Exposures:**

COVID-19 pandemic.

**Main Outcomes and Measures:**

Trends in trauma admission volume and injury patterns.

**Results:**

A total of 6777 patients in 2020 and 6937 in 2019 met inclusion criteria. Of those admitted in 2020, the median (interquartile range) age was 42 (28-61) years and 5100 (75.3%) were men. Mechanisms of injury were significantly different between the 2 years, with a higher incidence of penetrating trauma and fewer blunt injuries in 2020 compared with 2019 (penetrating: 1065 [15.7%] vs 1065 [15.4%]; blunt: 5309 [78.3%] vs 5528 [79.7%]). Overall admissions by interval in 2020 were 2681, 1684, and 2412, whereas in 2019 they were 2462, 1862, and 2613, respectively. There was a significant increase in overall admissions per week during the first interval (incidence rate ratio [IRR], 1.02; 95% CI, 1.002-1.04; *P* = .03) followed by a decrease in the second interval (IRR, 0.92; 95% CI, 0.90-0.94; *P* < .001) and, finally, an increase in the third interval (IRR, 1.05; CI, 1.03-1.07; *P* < .001). On subgroup analysis, blunt admissions followed a similar pattern to overall admissions, while penetrating admissions increased throughout the study period.

**Conclusions and Relevance:**

In this study, trauma centers throughout LAC experienced a significant change in injury patterns and admission trends during the COVID-19 pandemic. A transient decrease in volume was followed by a quick return to baseline levels. Trauma centers should prioritize maintaining access, capacity, and functionality during pandemics and other national emergencies.

## Key Points

**Question:**

How have trauma admission volume and injury patterns changed in metropolitan areas during the coronavirus disease 2019 pandemic?

**Findings:**

In this retrospective cohort study of 6777 trauma admissions in Los Angeles County from January 1 to June 7, 2020, overall volume transiently decreased but quickly returned to baseline. Mechanisms of injury were significantly different, with a steady increase in admissions for penetrating injuries.

**Meaning:**

These findings highlight the persistence of trauma burden in the community despite widespread restriction on public activity and the need to maintain trauma care resources and violence mitigation efforts during national emergencies.

## Teaser

This cohort study examines trends in trauma admissions throughout Los Angeles County, California, during the coronavirus disease 2019 (COVID-19) pandemic.

## Introduction

On January 20, 2020, the US Centers for Disease Control and Prevention reported the first confirmed case of coronavirus disease 2019 (COVID-19) in Washington state, and on January 26, 2020, public health officials confirmed the first case in Los Angeles County (LAC).^1,2^ In an effort to curb viral spread, local and state authorities across many areas of the country enforced social distancing guidelines with the closure of all nonessential businesses. California became the first state to implement a stay-at-home mandate by way of executive order on March 19, 2020.^3,4^ Despite these aggressive measures, by late July California had the highest number of cumulative cases, with LAC serving as the epicenter of the nation.^5^

While the pandemic has affected all facets of life, the nation’s health care infrastructure has been tested unlike during any event in recent history. The surge in patients with COVID-19 to emergency departments (ED), with many subsequently requiring admission to intensive care units (ICUs), forced many trauma centers to modify their existing protocols. The American College of Surgeons Committee on Trauma (ACS-COT) aptly put forth a guide on maintaining trauma center access and care during the pandemic.^6^ Its recommendations were directed toward resource allocation, preserving capacity, and ensuring adequate protection of trauma team members. This was released 1 day after California’s stay-at-home order was put into effect, at a time of great uncertainty in terms of how strict limitations would alter trauma volume or injury patterns.

To date, few studies have reported on how trauma care has been affected since the beginning of the pandemic.^7,8,9,10,11,12^ Early reports from the US and abroad indicate overall trauma volume decreased in the immediate period following stay-at-home orders or similar social distancing measures; however, many of these studies are based on single-institution data, and none appear to extend beyond the initial phase of the pandemic. What remains unclear is how sustained these trends have been, given that most cities have since loosened restrictions and reopened in stepwise fashion. LAC provides a unique setting for this analysis. In addition to being the most populous county in the nation, as of October 1, 2020, LAC had the highest number of confirmed COVID-19 cases and attributable deaths.^3,4^ The aim of this study was to evaluate the trends in LAC trauma center admissions during the course of the pandemic, including a countywide stay-at-home order and the early reopening period. We hypothesized that injury patterns would be different, while the number of trauma admissions would remain the same despite widespread public activity restrictions.

## Methods

### Study Design and Data Source

This retrospective cohort study was approved by the institutional review board at the University of Southern California. A waiver of informed consent was granted given the use of deidentified data. This study followed the Strengthening the Reporting of Observational Studies in Epidemiology (STROBE) reporting guideline.^13^ All data were obtained from the LAC Department of Health Services (DHS) Emergency Medical Services (EMS) Agency. The EMS Agency oversees all EMS and trauma center activity for the nearly 10.5 million residents of LAC. The EMS Agency’s Data Management Division is responsible for the collection, analysis, and dissemination of all EMS and inpatient data for trauma patients admitted at any of the 15 American College of Surgeons–verified level 1 and level 2 trauma centers within LAC.^14^ These data are organized in 2 separate registries. The EMS Provider registry is a collection of all prehospital clinical data. The Trauma registry is a collection of data from all trauma admissions at trauma centers within LAC, including those arriving by EMS or self-presentation. Data are submitted to the EMS Agency from each center within 60 days of patient discharge in accordance with Trauma Quality Improvement Program and National Trauma Data Bank submission requirements.^15^ For this study, the EMS Agency was queried for deidentified data from both registries.

### Study Eligibility

All trauma admissions (age ≥14 years) to the verified level 1 and level 2 trauma centers within LAC from January 1, 2020, to June 7, 2020 were included for analysis. This study period encompassed the first confirmed case of COVID-19 in the US, the implementation of a statewide stay-at-home order in California, and the initial stages of reopening (eTable in the Supplement). The same period from 2019 was used as historical control.

### Data Collection and Outcome Measures

We collected variables, including demographic characteristics, prehospital vital signs, injury data (mechanism, Abbreviated Injury Scale [AIS] by body region, injury severity score [ISS]), clinical data (ED vital signs, Glasgow Coma Scale [GCS], toxicology results, ED procedures performed), and patient outcomes (30-day mortality, incidence of acute respiratory distress syndrome [ARDS], hospital length of stay [HLOS], intensive care unit length of stay [ICU-LOS], and ventilator days). Race was self-reported or identified by a family member in accordance with LAC EMS Trauma Center Data Dictionary classifications and included in the study given the known disparities regarding COVID-19 and trauma.^15,16,17^ Mechanisms of injury were categorized as penetrating (gunshot wound, stab wound, or animal bite), other (electrical shock, hazardous material exposure, taser, thermal burn, or unknown), and blunt. *International Statistical Classification of Diseases and Related Health Problems, Tenth Revision (ICD-10) *codes were used to identify patients who underwent the following aerosol-generating procedures in the ED: intubation (codes 0BH17EZ and 0BH18EZ); tracheostomy or cricothyroidotomy (codes 0B113F4, 0B110F4, 0B110Z4, and 0B113Z4); thoracostomy tube placement, unilateral or bilateral (codes 0W9930Z, 0W9900Z, 0W9B30Z, and 0W9B00Z), and resuscitative thoracotomy (code 02JA0ZZ). Our primary outcome of interest was the trend in LAC trauma admissions. Secondary outcomes included mechanisms of injury, aerosol-generating procedures in the ED, HLOS, ICU-LOS, ventilator days, and 30-day mortality.

### Statistical Analysis

Descriptive statistics were used to summarize demographic characteristics, clinical data, injury data, and outcomes. Categorical variables were reported as numbers and percentages, and continuous variables were reported as medians with interquartile ranges (IQRs). HLOS, ICU-LOS, and ventilator days were reported as marginal means with 95% CIs. Univariate comparisons were made using the Wilcoxon rank-sum test for continuous variables and χ^2^ analysis for categorical variables. To capture changes in trauma admission volume between study periods, scatter plots with locally estimated smoothing (LOESS) were analyzed. A piecewise linear spline was then used for the final models. The plots for total admissions and blunt trauma admissions were each represented by 3 linear segments. In doing so, the study periods for these plots were divided into 3 statistical intervals. Knot positions were chosen visually from the LOESS plot and tested along with the 7 days before and after each position. Akaike information criterion (AIC) and Bayesian information criterion (BIC) were used for model comparison to find the best knot positions. Models with the lowest AIC and BIC were used for each final plot. Trends in admissions were reported as incidence rate ratios (IRRs) with 95% CIs. Two different multivariable regression models were used for secondary outcome analysis. Negative binomial regression was used to account for variance inflation in comparing HLOS, ICU LOS, and total ventilator days. Poisson regression with robust variance was used to compare ICU admission and 30-day mortality rates. A generalized estimation equation was applied in both models to account for hospital clustering. Overall, 176 of 13 714 patients (1.3%) were missing at least 1 covariate and were omitted from multivariable analysis. A 2-sided *P* < .05 was deemed statistically significant. Statistical analysis was performed using SAS version 9.4 (SAS Institute).

## Results

### Patient Characteristics

A total of 7201 trauma admissions were recorded during the study period in 2020 and 7381 during the same period in 2019. Following exclusion of patients younger than 14 years , 6777 patients (94.1%; median [IQR] age, 42 [28-61] years; 5100 [75.3%] men) in 2020 and 6937 (94.0%; median [IQR] age, 42 [28-60] years; 5112 [73.7%] men) in 2019 at 15 trauma centers were included for analysis (eFigure in the Supplement). Patient characteristics of each study period are outlined in Table 1. Median age was similar between groups (42 years). Median ISS was also similar; however, more patients experienced severe trauma in 2020 than in 2019 (ISS >15: 1338 [19.7%] vs 1276 [18.4%]; *P* = .04).

**Table 1.** Patient Characteristics by Year

Characteristic	Patients, No. (%)		*P* value
	Jan 1-Jun 7, 2019 (n = 6937)	Jan 1-Jun 7, 2020 (n = 6777)	
Age, median (IQR), y	42 (28-60)	42 (28-61)	.16
Sex			
Men	5112 (73.7)	5100 (75.3)	.02
Women	1825 (26.3)	1677 (24.7)	
Race			
White	2129 (30.7)	2013 (29.7)	.10
Black	1165 (16.8)	1096 (16.2)	
Hispanic	2844 (41.0)	2921 (43.1)	
Asian	417 (6.0)	359 (5.3)	
Other^a^	333 (4.8)	333 (4.9)	
Unknown	49 (0.7)	55 (0.8)	
Mechanism of injury			
Penetrating			.03
Total	1065 (15.4)	1065 (15.7)	
GSW	504 (7.3)	485 (7.2)	
SW	571 (8.2)	646 (9.5)	
Animal bite	23 (0.3)	24 (0.4)	
Blunt			
Total	5528 (79.7)	5309 (78.3)	
MVC	1574 (22.7)	1461 (21.6)	
MCC	606 (8.7)	589 (8.7)	
AVP	1512 (21.8)	1325 (19.6)	
Fall	1804 (26.0)	1811 (26.7)	
Assault	419 (6.0)	418 (6.2)	
Other	344 (5.0)	403 (6.0)	
AIS ≥3			
Head	1131 (16.3)	1119 (16.5)	.74
Chest	1006 (14.5)	994 (14.7)	.78
Abdomen	361 (5.2)	382 (5.6)	.26
Extremity	746 (10.8)	735 (10.9)	.86
ISS			
Median (IQR)	5 (2-13)	5 (2-13)	.95
ISS >15	1276 (18.4)	1338 (19.7)	.04
Field vital signs			
SBP <90 mm Hg	342 (5.2)	372 (5.8)	.17
HR >120 bpm	527 (8.0)	583 (9.0)	.04
GCS <9	537 (8.2)	535 (8.2)	.93
ED vitals			
SBP <90 mm Hg	249 (3.6)	250 (3.7)	.77
HR >120 bpm	572 (8.3)	623 (9.2)	.05
GCS <9	494 (7.2)	500 (7.4)	.58
Level 1 trauma center	3188 (46.0)	3207 (47.3)	.11
Positive alcohol screen, No./total No. (%)	1678/5544 (30.3)	1640/5360 (30.6)	.71
Positive toxicology screen, No./total No. (%)			
Cannabinoids	801/6913 (11.6)	988/6750 (14.6)	<.001
Amphetamines	560/6913 (8.1)	669/6750 (9.9)	<.001
Cocaine	227/6913 (3.3)	238/6750 (3.5)	.43

Abbreviations: AIS, Abbreviated Injury Scale; AVP, automobile vs pedestrian; bpm, beats per minute; ED, emergency department; GCS, Glasgow Coma Scale; GSW, gunshot wound; HR, heart rate; ISS, Injury Severity Score; MCC, motorcycle collision; MVC, motor vehicle collision; SBP, systolic blood pressure; SW, stab wound.

^a^ Other race includes Native American, Alaskan Native, Pacific Islander, Hawaiian, or any race not classified in the Los Angeles County Emergency Medical Services Trauma Data Dictionary.

There were significant differences in mechanism of injury between the 2 study periods. A higher incidence of penetrating injuries occurred in 2020 than in 2019 (1065 [15.7%] vs 1065 [15.4%]). Overall, blunt trauma decreased during the pandemic (5309 [78.3%] vs 5528 [79.7%]), with fewer automobile-pedestrian and motor vehicle collisions.

### Admission Trends

On overall trauma admission trend analysis, the study period was divided into 3 statistically defined intervals using knot positions of February 28 and April 9. In the first, second, and third intervals of 2020, there were 2681, 1684, and 2412 admissions, whereas in 2019 there were 2462, 1862, and 2613 admissions, respectively. The highest number of weekly admissions was 371 (50 penetrating, 297 blunt) in 2020 and 327 (48 penetrating, 251 blunt) in 2019. The lowest number of weekly admissions was 205 (37 penetrating, 155 blunt) in 2020 and 266 (33 penetrating, 222 blunt) in 2019.

**Figure 1.**
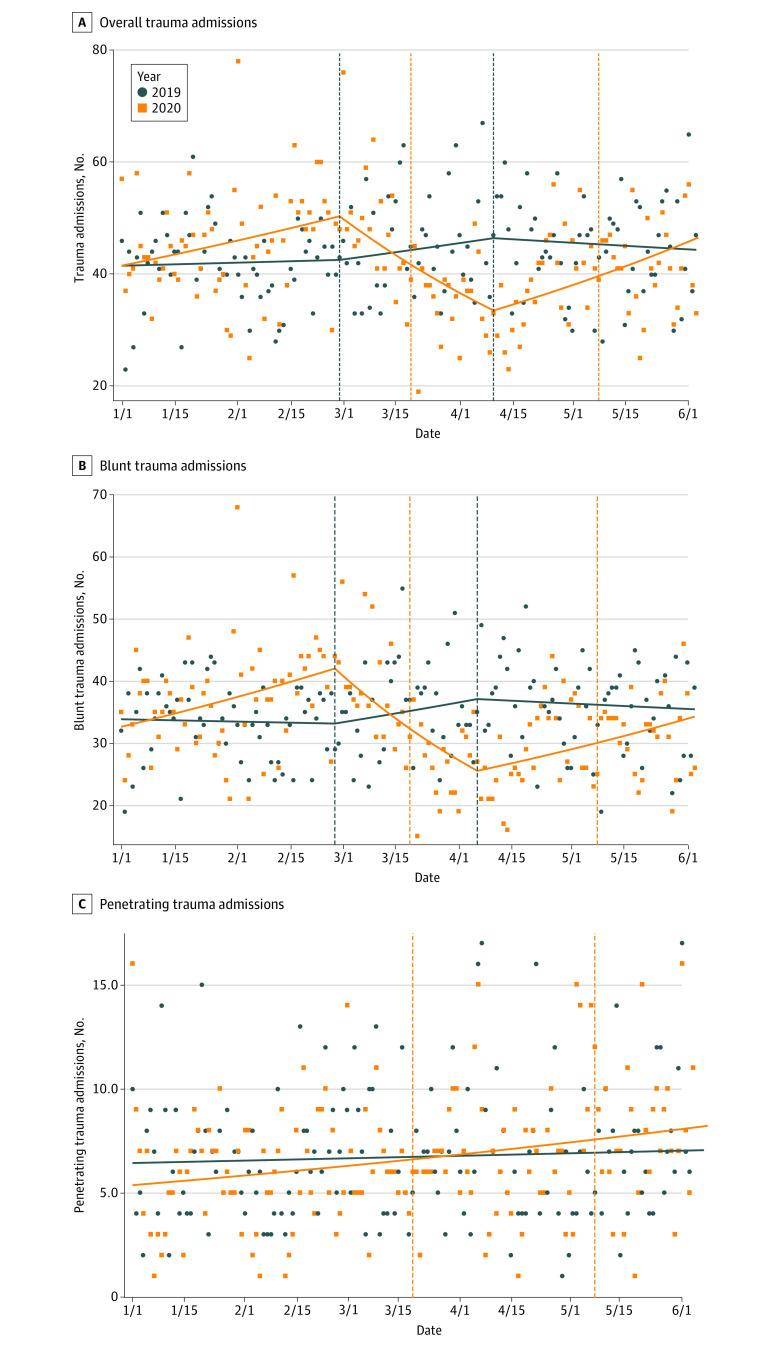
Trauma Admission Trends by Year Scatter plots with individual points representing daily countywide totals. Orange vertical lines represent implementation of a stay-at-home order (March 18) and the announcement of stage 2 of reopening (May 7). Blue vertical lines in panels A and B, which divide study period into 3 intervals, represent knot positions based on trend analysis.

Within the first interval, there was a significant increase in admissions per week in 2020 compared with 2019 (IRR, 1.02; 95% CI, 1.002-1.04; *P* = .03). This was followed by a significant decrease in the second interval (IRR, 0.92; 95% CI, 0.90-0.94; *P* < .001) and, finally, an increase in the third interval (IRR, 1.05; CI, 1.03-1.07; *P* < .001) (Figure 1A). In the subgroup analysis by injury mechanism, blunt trauma admissions were also divided into 3 statistically defined intervals using knot positions of February 27 and April 5. These per-week admissions followed a similar pattern to overall admissions in the first (IRR, 1.03; 95% CI, 1.01-1.06; *P* = .002), second (IRR, 0.89; 95% CI, 0.87-0.92; *P* < .001), and third intervals (IRR, 1.04; 95% CI, 1.02-1.06; *P* < .001), while penetrating trauma admissions per week increased throughout the study period (IRR, 1.01; 95% CI, 1.002-1.03; *P* = .03) (Figure 1B and C).

### Outcomes and Interventions

**Figure 2.**
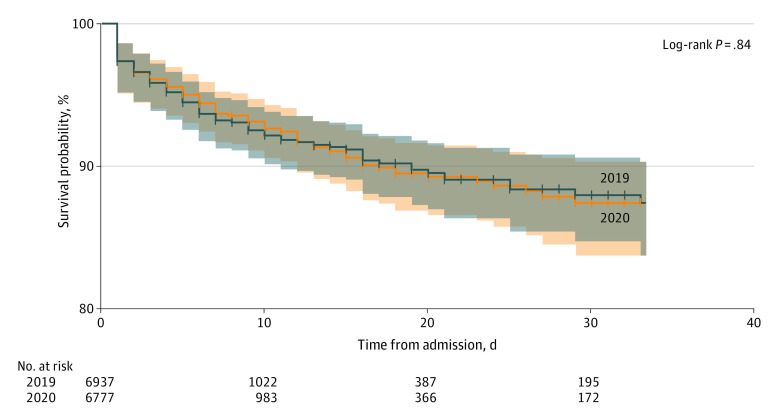
Kaplan-Meier Survival Curve Comparing 30-Day Mortality by Year Survival curve with 95% Hall-Wellner confidence bands for 30-day mortality stratified by year. Crosses indicate censored data, and shaded areas indicate 95% confidence bands.

There were no differences in mean HLOS or ventilator days (Table 2). The proportion of patients requiring ICU admission as well as the mean ICU-LOS were unchanged between the study periods. Fewer patients were diagnosed with ARDS in 2020 than in 2019 (7 [0.1%] vs 27 [0.4%]; *P* = .004). As shown in Figure 2, 30-day mortality was similar. The frequency of emergent aerosol-generating procedures performed in EDs also did not change significantly (Table 3).

**Table 2.** Patient Outcomes by Year

Variable	Mean (95% CI)		IRR or RR (95% CI)^a^	*P* value
	Jan 1-Jun 7, 2019	Jan 1-Jun 7, 2020		
Hospital LOS, d	5.49 (4.82-6.26)	5.29 (4.67-5.99)	0.96 (0.92-1.01)^b^	.15
Admission to ICU, No. (%)	2000 (28.8)	1930 (28.5)	0.96 (0.92-1.003)^c^	.07
ICU LOS, d^d^	5.53 (4.88-6.28)	5.41 (4.84-6.04)	0.98 (0.92-1.04)^b^	.48
Ventilator days^e^	1.55 (1.21-1.98)	1.44 (1.13-1.83)	0.93 (0.70-1.22)^b^	.59
30-d mortality, No. (%)	351 (5.1)	337 (5.0)	0.95 (0.84-1.06)^c^	.33

Abbreviations: ICU, intensive care unit; IRR, incident rate ratio; LOS, length of stay; RR, relative risk.

^a^ Variables used in the regression model were year, age by quartile (14-28, 29-42, 43-60, >60 years), sex, injury mechanism, injury severity score by quartile (0-2, 3-5, 6-13, >13), systolic blood pressure in emergency department (<90 mm Hg vs ≥90 mm Hg), heart rate in emergency department (>120 beats/min vs ≤120 beats/min), Glasgow Coma Scale in emergency department (<9 vs ≥9), trauma center (level 1 vs level 2).

^b^ IRR.

^c^ RR.

^d^ Applicable only to patients who were admitted to ICU.

^e^ Applicable only to patients who were ventilated.

**Table 3.** Patient Care Interventions by Year

ED procedure	Patients, No. (%)		*P* value
	Jan 1-Jun 7, 2019 (n = 6937)	Jan 1-Jun 7, 2020 (n = 6777)	
Intubation	644 (9.3)	635 (9.4)	.86
Thoracostomy tube placement^a^	311 (4.5)	312 (4.6)	.73
Surgical airway^b^	9 (0.1)	3 (0.04)	.10
Resuscitative thoracotomy	56 (0.8)	71 (1.1)	.14

Abbreviation: ED, emergency department.

^a^ Includes right, left, or bilateral.

^b^ Includes emergency tracheostomy or cricothyroidotomy.

## Discussion

In this retrospective cohort study, we observed unique epidemiologic trends in trauma that, to our knowledge, have not been reported in previous studies. On review of nearly 7000 trauma admissions in LAC, patient volume fluctuated significantly since the pandemic began. Compared with the year prior, admissions decreased surrounding the issuance of a stay-at-home order but returned to historical levels within weeks. Blunt trauma admissions mirrored the overall admission trend; however, penetrating trauma admissions maintained a slight upward trend throughout the pandemic. To our knowledge, this is the first population-level study to examine how trauma centers have been affected not only during the initial phase of the pandemic and lockdown period but also over the course of reopening.

In the first single-center report on the association of lockdown measures with trauma populations, Christey and colleagues^7^ reported a 50% decrease (71 vs 142; *P* < .001) in admissions to a level 1 trauma center in New Zealand in the 14 days following the declaration of a level 4 lockdown^18^—the highest level of restriction in their country—compared with the same time period in 2019. A similar study from Santa Clara County, California,^8^ showed a 79% decrease (81 vs 389; *P* < .001) in trauma activations in the 15 days after a countywide shelter-in-place order was put into effect compared with the 2 prior years. In their population, most activations were for blunt injuries, which was unchanged from their baseline data. Leichtle and colleagues^9^ published results from a level 1 trauma center in Richmond, Virginia, where trauma activations dropped 43% (mean [SD], 4.7 [2.6] activations vs 8.2 [0.3] activations) compared with historical control groups in the weeks after the March 17, 2020, declaration of a statewide public health emergency. Following the official stay-at-home order on March 30, 2020, they experienced a decline in violent trauma (10.6% vs 17%; *P* = .05) throughout the month of April. However, this was not the case in all areas of the country. Throughout multiple level 1 trauma centers in Philadelphia from March 9 to April 19, 2020, trauma contacts decreased 20% from what was seen in 2019 while penetrating injuries increased (34.5% vs 29.1%; *P* = .006).^10^

The initial drop in LAC trauma volume reflects what other groups have described. Interestingly, it appears this decline started in the weeks prior to the statewide stay-at-home order, perhaps a result of the governor’s State of Emergency declaration on March 4, 2020, as was seen in Virginia.^9^ The reason for this trend is likely multifactorial, and the initial decrease was largely anticipated, given the widespread restrictions and closure of all businesses deemed nonessential. Within California, traffic volume was reduced as much as 55% on state highways.^19^ Combined with general fear and anxiety surrounding the pandemic, this led to scenes of near empty metropolitan streets, which were depicted across various news outlets. As Forrester and colleagues^8^ pointed out, a similar drop in hospital admissions occurred after a severe acute respiratory syndrome outbreak in Toronto in 2003 and the Ebola virus outbreak in West Africa in 2014.^20,21^ Since the height of the pandemic, states have varied their approach in reopening. California outlined a 4-stage roadmap to reopening and announced progression from stage 1 to stage 2 on May 7, 2020. This allowed lower-risk workplaces, such as offices, retail shops, and manufacturing sites, to resume business with certain adaptations.^22^ We found an increase in trauma before restrictions were loosened, which leads us to believe that volume is not well associated with the degree of social distancing measures in place.

The increase in violent crime across LAC during this period coincides with what has taken place in other densely populated urban areas.^10^ Some have attributed this to heightened levels of stress caused by the socioeconomic impact of the pandemic.^10,11^ Combined with this has been a surge in the demand for guns. In March of this year, the US Federal Bureau of Investigation set the record for the highest number of firearm background checks by month since the bureau began keeping track in 1998.^23^ Shortly thereafter, the number in June surpassed this. Within California, these background checks increased by 42% from February to March.^24^ This pattern of public behavior is not uncommon after highly publicized tragedies or political shifts, with similar trends seen after the Sandy Hook mass shooting, the San Bernardino terrorist attack, and past presidential elections; however, unlike the current situation, these events were not associated with stay-at-home orders.^25^ Interestingly, Qasim and colleagues^10^ found a significant association between shooting events and neighborhoods most affected by COVID-19. They concluded that the social determinants of health contributing to high rates of transmission likely also contributed to violence. Along these lines, we must recognize that despite ongoing stay-at-home orders, many people do not comply with the intended social distancing.

Resource allocation was a focus of the ACS-COT guidelines, and trauma centers across LAC adjusted accordingly in collaboration with DHS.^6^ Admissions were expanded to rehabilitation facilities, and hospitals were able to transfer stable patients without COVID-19 to the docked Navy hospital ship, the USNS Mercy. Surgical ICUs often functioned as overflow medical ICUs. Outpatient staff were oriented and trained for deployment in ICU or medical-surgical units, while overall staff numbers were reduced to avoid exposure. Within each trauma bay, only essential personnel were present, and all patients were met with droplet precautions. This was crucial given the unchanged frequency of aerosol-generating procedures, which are often unavoidable because of the urgent circumstances during which they are performed. Patients requiring immediate operative intervention were routed to designated operating rooms reserved for those with positive or pending COVID-19 tests. Reports from across the globe in hard-hit areas similarly describe restructuring of their hospitals, policies, and staff.^26,27,28,29,30,31^ While several of these measures have proven successful, we advocate for ensuring the highest levels of trauma care and resources. Should the virus continue to spread unabated with another large surge in patients critically ill with COVID-19, elective procedures can once again be limited; however, the number of traumatic injuries requiring admission and ICU care may not change. In particular, penetrating trauma has been unfazed by social distancing measures and highlights the potential demand for operative capacity. For these reasons, we encourage regional health systems and trauma centers to anticipate these needs when planning for similar circumstances in the future.

### Limitations

Our study was inherently limited by its retrospective design and the use of registry data, which relies on accurate documentation in the medical charts and the registry itself. In addition, the registries did not include COVID-19–related variables, and this limited our ability to assess prevalence in the study population or its impact as a cause of death. Although most trauma patients in LAC are received at trauma centers, we were unable to obtain data on those who self-presented to hospitals without trauma center designation. The registry also limited our ability to capture suicidal injuries as a mechanism, which is an area that warrants further investigation. As we have seen from existing studies, the pandemic has affected trauma volume and injury patterns in different ways across various regions; therefore, the results of our analysis may not reflect global trends. In addition, seasonal variation in trauma between summer and winter months likely had a confounding effect. Along these lines, the amount of rainfall and the number of wildfires differed between years and potentially affected our results.^32,33^ It is worth noting that our study period also included the deaths of Breonna Taylor and George Floyd, 2 African American citizens whose murders sparked nationwide unrest and protests. The degree to which this contributed to our findings remains unclear. Nonetheless, we provide insight from the nation’s most populous county to the growing collection of reports from trauma systems across the world during this pandemic.

## Conclusions

LAC has been hit hard by the COVID-19 pandemic, as have several other areas throughout the nation and across the world. In this study, trauma volume in LAC decreased surrounding a stay-at-home order but quickly returned to baseline historical rates. Furthermore, trauma admissions maintained their need for resource-heavy levels of care.

## Notes

Supplement. **eTable.** Timeline of COVID-19 Pandemic Within Los Angeles County **eFigure.** Volume of Admissions to Trauma Centers in Los Angeles County by Year
