# Supplementary material for: Trends in Trauma Admissions During the COVID-19 Pandemic in Los Angeles County, California
Source: JAMA Netw Open. 2021 Feb 22;4(2):e211320. doi: 10.1001/jamanetworkopen.2021.1320 (PMC7900857; doi:10.1001/jamanetworkopen.2021.1320)
Supplement: Supplement. — eTable. Timeline of COVID-19 Pandemic Within Los Angeles County eFigure. Volume of Admissions to Trauma Centers in Los Angeles County by Year [file jamanetwopen-e211320-s001.pdf]

Supplemental Online Content

Ghafil C, Matsushima K, Ding L, Henry R, Inaba K. Trends in trauma admissions during the COVID-19 pandemic in Los Angeles County, California. *JAMA Netw Open*. 2021;4(2):e211320. doi:10.1001/jamanetworkopen.2021.1320

eTable. Timeline of COVID-19 Pandemic Within Los Angeles County

eFigure. Volume of Admissions to Trauma Centers in Los Angeles County by Year

This supplemental material has been provided by the authors to give readers additional information about their work.

© 2021 Ghafil C et al. *JAMA Network Open.*

eTable. Timeline of COVID-19 Pandemic Within Los Angeles County

Date	Event
January 17, 2020	CDC initiates enhanced screenings at LAX
January 26, 2020	LAC confirms first case
March 4, 2020	LAC declares a local health emergency
March 12, 2020	Cancellation of all non-essential public events and events on city-owned properties with 50 or more people
March 16, 2020	Closure of Los Angeles Unified School District
March 19, 2020	Statewide stay-at-home order implemented
April 14, 2020	LAC cases top 10,000, with 360 related deaths
May 7, 2020	Governor announces state will enter stage 2 of its reopening roadmap
June 1, 2020	LAC cases top 60,000

CDC, Centers for Disease Control and Prevention; LAX, Los Angeles International Airport; LAC, Los Angeles County.

© 2021 Ghafil C et al. *JAMA Network Open.*

eFigure. Volume of Admissions to Trauma Centers in Los Angeles County by Year

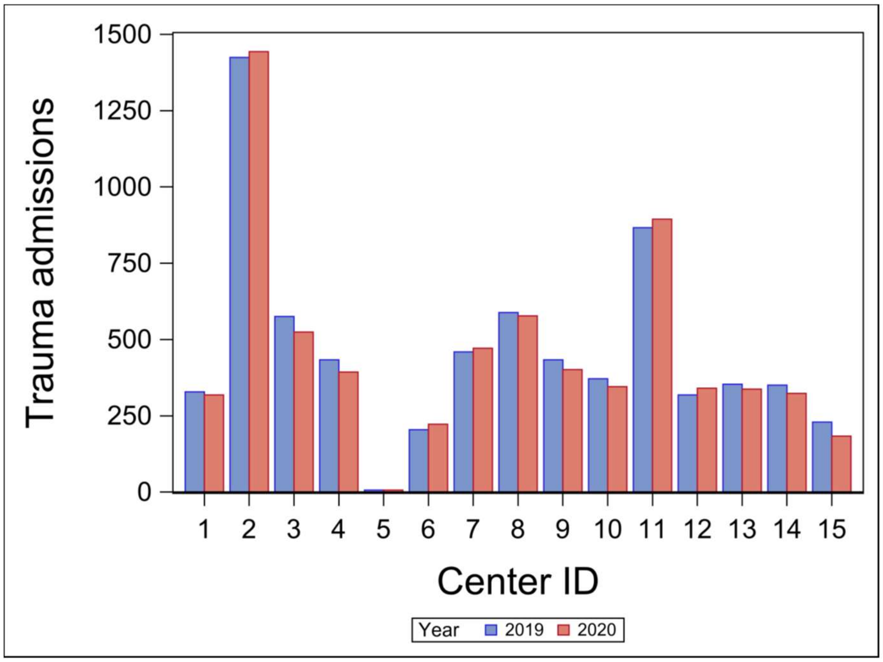

© 2021 Ghafil C et al. *JAMA Network Open.*
